# Serum C-reactive protein to albumin ratio as a novel inflammation biomarker in psoriasis patients treated with adalimumab, ustekinumab, infliximab, and secukinumab: a retrospective study

**DOI:** 10.3325/cmj.2020.61.333

**Published:** 2020-08

**Authors:** Funda Tamer, Emine Avcı

**Affiliations:** 1 Department of Dermatology, Gazi University School of Medicine, Ankara, Turkey; 2 Department of Infectious Diseases, General Directorate of Public Health, Ankara, Turkey

## Abstract

**Aim:**

To assess C-reactive protein to albumin ratio (CAR) before and after treatment with biological agents in patients with psoriasis to determine whether CAR can be used as an inflammation biomarker.

**Methods:**

Medical records of patients with psoriasis treated with biological agents at the Department of Dermatology, Gazi University Hospital were retrospectively evaluated between June 2018 and August 2019. The patients were divided into four groups based on the type of treatment (adalimumab, ustekinumab, infliximab, secukinumab). CAR was evaluated before and three months after treatment.

**Results:**

The study enrolled 157 patients with psoriasis vulgaris (91 male) aged between 18 and 85. CAR significantly decreased in all treatment groups (adalimumab group *P* < 0.001; ustekinumab *P* = 0.006; infliximab *P* = 0.007; secukinumab *P* < 0.001). The most prominent decrease in CAR was observed in patients treated with secukinumab (median CAR before treatment 1.52 [1.01-3.04] and after treatment 0.84 [0.62-0.99]).

**Conclusion:**

CAR may be a good indicator of systemic inflammation in psoriasis patients treated with biological agents.

Psoriasis is a multisystem disease characterized by chronic inflammation (1-3). About 11%-40% of patients with psoriasis suffer from psoriatic arthritis (4). Furthermore, patients with psoriasis are prone to various systemic comorbidities, such as hypertension, diabetes mellitus, hyperlipidemia, metabolic syndrome, stroke, cardiovascular disorder, and inflammatory bowel disease as a result of systemic inflammation (4). The extent of cutaneous lesions and relationship between the skin involvement and comorbid conditions depend on the degree of systemic inflammation (1). Inflammation in psoriasis patients is effectively treated with biological agents – tumor necrosis factor-α (TNF-α) inhibitors such as infliximab, adalimumab, and etanercept; ustekinumab (interleukin [IL]-12/23 inhibitor), and secukinumab (IL-17 inhibitor) (4).

However, 20%-30% of the patients with psoriasis do not respond to biological therapy (5). Since inflammatory state indicates disease severity, there is a need for inflammation biomarkers that would facilitate prognosis making, selection of appropriate individual treatment, and treatment efficacy evaluation. Inflammation biomarkers that have been previously used in psoriasis patients include serum levels of C-reactive protein (CRP), haptoglobin, complement 3 (C3), C4, TNF-α, IL-6, IL-8, IL-12, elafin, neutrophil-lymphocyte ratio (NL), and platelet-lymphocyte ratio (PL) (5-7).

Recently, CRP to albumin ratio (CAR) has been proposed as an inflammation biomarker and a prognostic factor in inflammatory processes such as cardiovascular disease and ischemic stroke (8,9). CAR indicates inflammation more efficiently than isolated serum CRP levels (9,10). Although it has been considered as a valuable indicator of inflammatory status, CAR has not been evaluated in patients with psoriasis. Therefore, this study assessed CAR before and after treatment with biological agents: adalimumab, ustekinumab, infliximab, and secukinumab, in patients with psoriasis.

## PATIENTS AND METHODS

We retrospectively reviewed the medical records of 157 patients with psoriasis vulgaris who were treated with adalimumab, ustekinumab, infliximab, and secukinumab at the Department of Dermatology of Gazi University Hospital between June 2018 and August 2019. Data on age, sex, treatment agent, coexisting psoriatic arthritis, serum biochemistry profile, including serum CRP and albumin levels, were collected. The study enrolled all patients aged 18 and over who had available data. The exclusion criteria were pregnancy, lactation, malignancy, chronic inflammatory disorders, such as lupus, rheumatoid arthritis, inflammatory bowel disease, liver failure, chronic renal diseases, and malnutrition. The study was approved by Gazi University Ethics Committee (2019-309).

CAR was calculated as the ratio of serum CRP level (mg/L) to serum albumin level (g/dL), which were obtained from biochemistry profile. The patients were divided into four groups based on the type of biological agent they received (adalimumab, ustekinumab, infliximab, secukinumab). Serum CAR was evaluated in all patients before and three months after the initiation of biological agent treatment. In addition, serum CAR was evaluated in each treatment group before and three months after biological agent treatment. Serum CAR was also compared between the treatment groups.

### Statistical analysis

Data are expressed as mean ± standard deviation or median and interquartile range for quantitative variables, and counts and percentage for categorical variables. The normality of distribution was tested with the Kolmogorov-Smirnov test or Shapiro-Wilk test. The significance of differences in CAR before and after treatment was assessed with the Mann-Whitney U test or Wilcoxon signed rank test for continuous variables and χ^2^ test for categorical variables. The significance of differences between treatment groups was evaluated with the Kruskal-Wallis analysis of variance for continuous variables. *P* < 0.05 was considered significant. Statistical analysis was performed with SPSS, version 20.0. (IBM Corp., Armonk, NY, USA).

## RESULTS

The study enrolled 157 patients with psoriasis vulgaris (91 or 58% male). The mean age was 48.33 ± 12.5 years (range, 18-85). Thirty-one (19.7%, 18 male) patients were previously diagnosed with psoriatic arthritis, with no significant difference in the number of male and female patients. Sixty-five (41.4%) patients were treated with adalimumab, 42 (26.8%) with ustekinumab, 32 (20.4%) with infliximab, and 18 (11.5%) with secukinumab.

The median CAR was 0.83 (0.53-1.45) in women and 1.03 (0.65-1.79) in men (*P* = 0.11). The median CAR before treatment was 1.01 (0.66-1.01) in patients with psoriatic arthritis and 0.95 (0.58-1.59) in patients without psoriatic arthritis (*P* = 0.41). The median CAR three months after treatment was 0.80 (0.40-1.38) in patients with psoriatic arthritis and 0.72 (0.47-1.06) in patients without psoriatic arthritis (*P* = 0.48). Decrease in CAR after treatment was significant both in patients with arthritis (*P* = 0.002) and patients without arthritis (*P* < 0.001).

The median CAR in all patients before and three months after treatment was 0.97 (0.61-1.61) and 0.73 (0.45-1.11), respectively (*P* < 0.001). The median CAR also significantly decreased three months after the initiation of adalimumab, ustekinumab, infliximab, and secukinumab (*P* < 0.001, *P* = 0.006, *P* = 0.007, *P* < 0.001, respectively) (Table 1).

**Table 1:** The median serum C-reactive protein to albumin ratio (CAR) before and three months after biological agent treatment

	CAR median (interquartile range)			
	before treatment		after treatment	P
Adalimumab (n = 65)		0.79 (0.52-1.14)	0.62 (0.36-1.02)	<0.001
Ustekinumab (n = 42)		0.90 (0.63-1.73)	0.82 (0.56-1.34)	0.006
Infliximab (n = 32)		1.08 (0.64-1.85)	0.84 (0.48-1.23)	0.007
Secukinumab (n = 18)		1.52 (1.01-3.04)	0.84 (0.62-0.99)	<0.001
All patients (n = 157)		0.97 (0.61-1.61)	0.73 (0.45-1.11)	<0.001
Patients with arthritis (n = 31)		1.01 (0.66-1.01)	0.80 (0.40-1.38)	0.002
Patients without arthritis (n = 126)		0.95 (0.58-1.59)	0.72 (0.47-1.06)	<0.001

The median CAR before treatment was highest in the secukinumab group (1.52 [1.01-3.04]), followed by infliximab, ustekinumab, and adalimumab group. Before treatment, the median CAR was significantly higher in the secukinumab compared with adalimumab group (*P* = 0.001). After treatment, the median CAR was highest in the infliximab group (0.84 [0.48-1.23]), followed by the secukinumab, ustekinumab and adalimumab group. However, after treatment, the median CAR did not significantly differ between the treatment groups (*P* = 1.09).

Change in median CAR after treatment was 0.76 (0.33-1.42) in the secukinumab, 0.14 (-0.07-0.47) in infliximab, 0.12 (-0.01-0.49) in ustekinumab, and 0.10 (-0.01-0.32) in adalimumab group. The most prominent decrease in CAR was detected in the secukinumab group (*P* < 0.001).

## DISCUSSION

In this study, the median CAR significantly decreased in psoriasis patients three months after biological agent treatment. Furthermore, CAR decreased significantly in all treatment groups. These results indicate that CAR may be used as a novel and easily obtainable inflammation biomarker in psoriasis patients treated with biological agents.

In our study, 31 (19.7%) patients had psoriatic arthritis. Psoriatic arthritis is a type of systemic inflammation related to psoriasis. CAR was similar in patients with and without psoriatic arthritis, and in patients with psoriatic arthritis it decreased significantly three months after treatment. These results indicate that in addition to being an inflammation biomarker of psoriasis, CAR might be used as an inflammation biomarker of psoriatic arthritis in psoriasis patients treated with biological agents.

CRP is a widely used indicator of systemic inflammation (6). High serum CRP levels have been reported in patients with active psoriasis and chronic plaque psoriasis (2). Serum CRP levels and complete blood count-derived biomarkers, such as NL and PL, have been associated with disease severity and joint involvement in patients with psoriasis. Serum NL and PL levels decrease in psoriasis patients after infliximab, adalimumab, and ustekinumab treatment similarly to serum CRP levels (6).

Recently, CAR has become accepted as an inflammation marker in various disorders. It has been used to detect inflammatory process and predict the prognosis of pancreatic cancer, colorectal cancer, coronary artery disease, and acute ischemic stroke (9,11-14). CAR is calculated by proportioning serum CRP levels to albumin, which are both acute phase reactants (10). However, CAR might indicate inflammation more successfully than isolated serum CRP levels or albumin levels alone (9,10).

The risk of cardiovascular disease in psoriasis patients might be decreased by biological agents treatment since inflammation is the main etiological factor of atherosclerosis (8,15-17). In this study, the most prominent decrease in CAR was observed in patients treated with secukinumab. Secukinumab exerts its anti-inflammatory effect by targeting IL-17, a cytokine that plays an essential role in both the etiology of chronic skin inflammation in psoriasis and the etiology of atherosclerosis. Moreover, secukinumab may improve the endothelial function in psoriasis patients by reducing blood oxidative stress. Therefore, it has been proposed to play a role in preventing psoriasis comorbidities (18-20).

The limitations of this study are retrospective design and lack of data about the relationship between CAR values and psoriasis severity. Regardless of these limitations, this is the first study that evaluated CAR in psoriasis. Since CAR decreased significantly in patients with psoriasis after adalimumab, ustekinumab, infliximab, and secukinumab treatment, we suggest that it can be used as a novel, quickly and easily obtainable inflammation biomarker in psoriasis patients treated with biological agents.
